# Variation in tree mortality and regeneration affect forest carbon recovery following fuel treatments and wildfire in the Lake Tahoe Basin, California, USA

**DOI:** 10.1186/1750-0680-7-7

**Published:** 2012-06-28

**Authors:** Chris H Carlson, Solomon Z Dobrowski, Hugh D Safford

**Affiliations:** 1 Department of Forest Management, University of Montana, Missoula, MT, 59812, USA; 2 USDA Forest Service, Pacific Southwest Region, Vallejo, CA, 94592, USA; 3 Department of Environmental Science and Policy, University of California, Davis, CA, 95616, USA

**Keywords:** Wildfire, Fuel reduction treatment, Thinning, Forest carbon, Tree mortality, Tree regeneration, Sensitivity analysis, Forest Vegetation Simulator

## Abstract

**Background:**

Forest fuel treatments have been proposed as tools to stabilize carbon stocks in fire-prone forests in the Western U.S.A. Although fuel treatments such as thinning and burning are known to immediately reduce forest carbon stocks, there are suggestions that these losses may be paid back over the long-term if treatments sufficiently reduce future wildfire severity, or prevent deforestation. Although fire severity and post-fire tree regeneration have been indicated as important influences on long-term carbon dynamics, it remains unclear how natural variability in these processes might affect the ability of fuel treatments to protect forest carbon resources. We surveyed a wildfire where fuel treatments were put in place before fire and estimated the short-term impact of treatment and wildfire on aboveground carbon stocks at our study site. We then used a common vegetation growth simulator in conjunction with sensitivity analysis techniques to assess how predicted timescales of carbon recovery after fire are sensitive to variation in rates of fire-related tree mortality, and post-fire tree regeneration.

**Results:**

We found that fuel reduction treatments were successful at ameliorating fire severity at our study site by removing an estimated 36% of aboveground biomass. Treated and untreated stands stored similar amounts of carbon three years after wildfire, but differences in fire severity were such that untreated stands maintained only 7% of aboveground carbon as live trees, versus 51% in treated stands. Over the long-term, our simulations suggest that treated stands in our study area will recover baseline carbon storage 10–35 years more quickly than untreated stands. Our sensitivity analysis found that rates of fire-related tree mortality strongly influence estimates of post-fire carbon recovery. Rates of regeneration were less influential on recovery timing, except when fire severity was high.

**Conclusions:**

Our ability to predict the response of forest carbon resources to anthropogenic and natural disturbances requires models that incorporate uncertainty in processes important to long-term forest carbon dynamics. To the extent that fuel treatments are able to ameliorate tree mortality rates or prevent deforestation resulting from wildfire, our results suggest that treatments may be a viable strategy to stabilize existing forest carbon stocks.

## Background

As society attempts to manage forests as sinks to offset anthropogenic increases in atmospheric carbon, there has been an effort to understand how human and natural disturbances impact forest carbon stocks at spatial and time scales important to carbon sequestration. Some disturbances are completely outside or completely within the control of humans (i.e., drought or land use change), but wildfires are both: they are responsive to management decisions such as fire suppression or fuels manipulation, but many of the factors that influence fire regimes (ignitions, climate or weather) and the corresponding impacts on forest carbon resources remain beyond our control or prediction. It has been postulated that forest fuel reduction treatments (which typically include some combination of tree removal or prescribed burning) may protect or stabilize forest carbon stocks in wildfire-prone forests, if they successfully reduce future wildfire severity [1-5]. However, these treatments also reduce forest biomass and therefore forest carbon storage [2,6]. This sets up an inherent tension between carbon storage and fuel treatments that has been the focus of recent debate [3,4,7-10]. We have a great deal of observational and experimental evidence for the short-term impacts of wildfire and fuel management on forest carbon budgets [2,6,11-15] but investigations into long-term carbon dynamics following treatment and wildfire have necessarily relied on simulation models [7-9,16]. However, many of these long-term simulations do not examine how natural variation in important ecosystem dynamics, such as wildfire severity and post-fire vegetation recovery, might affect expectations whether fuel treatments could protect or stabilize forest carbon stocks [17].

Fuel reduction treatments are widely used management tools that allow us to modify wildfire behavior and reduce the potential for stand replacing fire [18]. Although there have been cases where fuel treatments do not reduce the severity of fire due to extreme fire weather, insufficient removal of fuels, small treatment units, or vegetation growth since treatment [19,20], fuel treatments have been shown to reduce fire severity and rates of tree mortality when management sufficiently reduces surface, ladder, and canopy fuels [21,22]. In terms of carbon, North and Hurteau [5] observed reductions in fire severity and wildfire emissions in stands where 18–33% of aboveground carbon was removed during treatments completed 5 years before wildfire. This range of biomass removal rates is similar to those reported in studies where fuel treatments successfully reduced simulated wildfire effects [2,23,24]. Treated stands are thought to maintain similar or smaller total forest carbon stocks than do untreated stands immediately after wildfire, because fuel treatments often remove more carbon than is saved through reductions in pyrogenic emissions, which limits the perceived short-term carbon benefit of forest fuel management activities [5,9]. When treatments successfully reduce fire severity, they maintain a higher proportion of carbon as live vegetation following fire, suggesting that the potential carbon benefit of fuel treatments may be realized on a longer time scale, as fire-killed trees in severely burned stands continue to emit carbon and surviving or regenerating vegetation continues to sequester carbon [3,5,14,15].

Over longer time periods, forest carbon storage is controlled by the balance between carbon accumulation through photosynthesis, carbon loss through decay, and offsite removal or non-biological carbon emissions including pyrogenic emissions (Net Ecosystem Production, NEP, [25]). Fuel treatments will only be able to promote additional carbon storage if they cause NEP to be more positive over a long time period as compared to untreated stands. Over a fire return interval, NEP will be largely governed by direct carbon losses from wildfire or fuel treatments, indirect emissions as fire-killed trees decay, and by the growth of surviving and regenerating vegetation [3,26]. Fire simulators such as FVS-FFE [27] use well-established empirical models to predict first order fire effects [28]. However, a number of stochastic factors (such as fire weather, fuel conditions or ignition timing) make prediction of specific fire effects difficult [29]. Fuel treatments may fail to reduce fire severity and treatment efficacy is known to decline with time [20]. Like wildfire effects, post-wildfire regeneration may be difficult to predict. Previous research has shown that post-fire regeneration patterns may be highly temporally and spatially variable along gradients of disturbance severity, species characteristics, climate, microsite conditions, and competitive factors [30-34]. Although wildfires may promote the regeneration of fire-adapted species, severe wildfires may cause temporary or permanent shifts in the structure or composition of forest communities [32,35-37]. Predictions of long-term forest carbon storage after wildfire which do not take into account uncertainties in important ecosystem processes that affect rates of carbon accumulation (such as mortality or regeneration) may contribute to the controversy over the carbon costs and benefits of fuel treatments without producing results that are transferrable to management [5,17].

In this study, we use information collected from fuel treated and untreated stands that burned in a natural mixed severity wildfire, along with a commonly used vegetation simulator to address the following questions: 1) What was the impact of fuel treatments and wildfire on aboveground carbon storage in treated and untreated stands in our study area?; 2) How long will treated and untreated stands take to recover pre-disturbance carbon storage?; and more generally, 3) How is forest carbon recovery after wildfire sensitive to variation in fire-related tree mortality and rates of post-fire tree regeneration? By answering these questions, we hope to provide context to how natural variability in wildfire severity and post-wildfire recovery might influence the ability of fuel treatments to protect forest carbon storage.

### Methods overview

We collected vegetation, mortality, and regeneration data in treated (“Treated Burned”; TB) and untreated (“Not Treated Burned”; NTB) forest stands located in a mixed-conifer forest in the central Sierra Nevada, California, U.S.A, that burned in a 2007 wildfire. We used these data in conjunction with the Western Sierra variant of the Forest Vegetation Simulator (FVS) [38] to estimate forest carbon stocks and to simulate forest growth processes. FVS is an individual-tree, distance independent, growth and yield model that is widely used by academic and agency researchers investigating how management, disturbance, and climate change affect forest carbon storage [39-41]. We estimated the size of five aboveground carbon pools in TB and NTB stands (live trees, dead trees, coarse woody debris, fine woody debris, litter and duff) in our study area before and after thinning and wildfire, in order to characterize the pre- and post-disturbance aboveground carbon storage and fluxes due to disturbance (Table 1). We then used FVS to simulate vegetation growth after fire to compare timescales of carbon recovery after fire between TB and NTB stands, and assess how differences between modeled and observed estimates of tree mortality influenced recovery timing in our study area. Finally, we used observations of mortality and regeneration rates acquired over three years at our study site to bound the range of potential fire effects and regeneration trajectories in our study area to use as inputs to a sensitivity analysis. In our sensitivity analysis, we assessed how the timing of carbon recovery after fire is sensitive to variation in rates of fire-related tree mortality and post-fire regeneration. We used two baselines (pre- and post-fuel treatment aboveground carbon storage) to estimate recovery timing.

**Table 1:** Methods used to estimate carbon density before and after disturbance by treatment and wildfire

			**Timestep**				
**C Pool**	**Source of Biomass equation**	**Biomass to C factor**	**Pre-Treatment**	**Pre-Wildfire**	**2008**	**2009**	**2010**
**Live trees**	FVS-WS default [38]	0.5	Prefire live treelist plus stumps	Prefire live treelist	As observed 2008	As observed 2009	As observed 2010
**Dead Trees**	FVS-WS default [38]	0.5	Prefire snag list	Prefire snag list	“	“	“
**Wood > 7.62 cm**	Waddell et al. [42]	0.5	Surface C pools: Average from untreated stands outside fire	Surface C: pools Average from untreated **or** treated stands outside fire	“	“	“
**Wood < 7.62 cm**	Brown [43], van Wagtendonk [44]	0.5			“	“	“
**Litter and duff**	van Wagtendonk [45]	0.37 [46]			“	“	“

Methods used to calculate carbon density for five aboveground pools (live trees, dead trees, small woody debris, large woody debris, and litter and duff) at five time steps on 13 treated and 26 untreated Common Stand Exam plots in the Angora fire.

## Results

### Short-term impact of treatment and wildfire on C pools

Before disturbance by fuel treatments or wildfire, treated and untreated stands in our study site stored comparable amounts of aboveground carbon (183.2 and 175.89 Mg C ha-1 respectively, Wilcoxon rank sum test p-value = 0.758, Table 2). In treated stands, carbon losses due to fuel treatment (tree removal and pile burning) totaled 70.48 Mg ha-1 (or 38% of aboveground C). We estimate that roughly 40% of C losses during treatment were due to tree removal (28.3 Mg C ha-1), and 60% to pile burning (values based on observations of stumps and the difference in average surface fuel loads between treated and untreated plots outside the fire [42.15 Mg C ha-1]). Before wildfire and after fuel treatments, treated stands stored significantly less aboveground carbon than untreated stands (111.85 and 175.51 Mg C ha-1, respectively, Wilcoxon rank sum test p-val = 0.0002). After wildfire, TB and NTB stands stored similar amounts of total aboveground carbon (89.27 and 101.08 Mg C ha-1, respectively, rank sum test p-val > 0.5). Post-wildfire estimates of total aboveground C storage were 22.58 and 74.43 Mg C ha-1 lower than pre-fire estimates in TB and NTB stands respectively, suggesting overall pyrogenic emissions of 20% to 42% (Table 2).

**Table 2:** Estimates of carbon density in treated and untreated stands

		**Carbon density (Mg C ha**^**-1**^**)**			
**Time Step**	**Pool**	**TB stand (n = 13)**	**NTB stand (n = 26)**	**p value**	**Significance**
Pre-treatment	Live Tree C	108.20	96.43	0.471	
	Snag C	3.06	8.39	0.028	**
	FWD C**†**	3.68	3.68	n.a.	n.a.
	CWD C**†**	33.87	33.87	n.a.	n.a.
	Floor C**†**	33.52	33.52	n.a.	n.a.
	Aboveground C	182.33	175.89	0.758	
Pre-fire	Live Tree C	79.89	96.05	0.489	
	Snag C	3.06	8.39	0.028	**
	FWD C**‡**	2.22	3.68	0	*
	CWD C**‡**	3.85	33.87	0.019	**
	Floor C**‡**	22.82	33.52	0.258	
	Aboveground C	111.85	175.51	0.000	***
2008	Live Tree C	57.94	10.82	0.000	***
	Snag C	15.34	70.99	0.000	***
	FWD C	1.20	0.61	0.087	*
	CWD C	2.01	10.62	0.010	**
	Floor C	12.78	8.03	0.003	***
	Aboveground C	89.27	101.08	0.691	
2009	Live Tree C	53.27	7.66	0.000	***
	Snag C	18.51	73.65	0.000	***
	FWD C	1.66	1.11	0.159	
	CWD C	3.15	11.12	0.025	**
	Floor C	13.02	8.53	0.038	**
	Aboveground C	89.61	102.06	0.607	
2010	Live Tree C	49.59	6.93	0.000	***
	Snag C	20.33	74.28	0.000	***
	FWD C	1.81	2.16	0.368	
	CWD C	4.97	14.81	0.003	***
	Floor C	13.42	7.37	0.009	***
	Aboveground C	90.11	105.55	0.586	

Estimates of carbon density for five aboveground C pools (live trees, snags, Fine Woody Debris [<7.62 cm diameter], Coarse Woody Debris [> = 7.62 cm diameter] and forest floor [litter and duff combined]) in treated (TB) and untreated (NTB) stands in the Angora fire before disturbance by treatment and wildfire, and for three years after wildfire. **†**: Pre-treatment carbon densities of surface fuels are assumed to be the same in TB and NTB plots **‡**: Carbon densities of surface fuels before fire were estimated from 9 treated and 9 untreated plots outside the wildfire.

**Table 3:** Tree regeneration rates in treated and untreated stands in the Angora fire

**Estimate**	**Statistic**	**Treated plots (n = 37)**	**Untreated plots (n = 71)**
Total Natural	Mean	794.74	2765.14
Seedlings ha-1	Sd	893.66	13946.16
	Median	518.92	0
Total Planted	Mean	65.45	151.05
Seedlings ha-1	Sd	255.75	292.28
	Median	0	0

Rates of tree regeneration (seedlings ha-1) observed in treated and untreated stands at our study site three years after wildfire (2010) without distinguishing between species or year of establishment.

Although total post-fire C storage did not differ between treatments, untreated stands stored significantly more carbon in non-living pools (snags and CWD). Carbon contained by dead trees and coarse woody debris represents 84% of all aboveground C in untreated stands in 2010 vs. 28% in treated stands. Likewise, treated stands maintained more live tree carbon after fire. Treated stands retained 55% of aboveground C as live tree C in 2010, while untreated stands maintained 6.5% aboveground C as live trees in 2010. We observed continued tree mortality throughout the three years of our study. Mortality occurring within one year of fire (through 2008) represented 73% and 96% of all live tree C that died as a result of fire in treated and untreated stands, respectively, with the remaining 27% and 4% dying two and three years after fire.

From regeneration plots surveyed in treated and untreated stands (n = 37 and n = 71, respectively), we found that treated stands have lower mean seedling densities than untreated stands (794.74 vs. 2765.14 natural seedlings ha-1, Table 3) three years after fire. However, median rates of regeneration in treated stands are higher than those in untreated stands (518.93 vs. 0 seedlings ha-1), as 51% of plots in untreated stands had no natural tree regeneration three years after fire, vs. only 14% of plots in treated stands (see Additional file 1 for data regarding carbon pool sizes, regeneration rates, and predicted and observed mortality rates at 39 CSE plots).

### Long-term impact of treatment and wildfire on C pools

**Figure 1:**
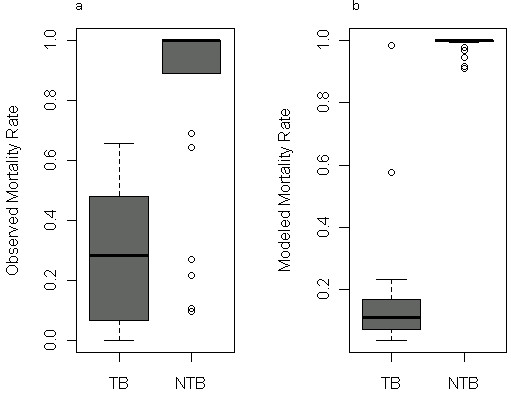
**Rates of tree mortality by treatment, observed vs. simulated mortality.** Rates of mortality (proportion basal area killed) estimated using (**a**) field based observations and (**b**) the Forest Vegetation Simulator Fire and Fire Effects extension [27] to predict tree mortality, for plots located in treated (TB) and untreated (NTB) stands at our study site. See Additional file 2 for details regarding wildfire simulation.

Three years after wildfire, we observed that TB stands in the Angora fire experienced lower rates of fire-related tree mortality than NTB stands (mean 31% [sd 24%] vs. mean 84% [30%] basal area mortality, respectively, Figure 1a). In comparison, rates of mortality predicted by FVS-FFE were lower than observed rates in TB stands (predicted mean 21% [27%] vs. observed 31% BA mortality) and higher than observed rates in NTB stands (predicted 99% [2%] vs. observed 84% BA mortality, Figure 1b).

**Figure 2:**
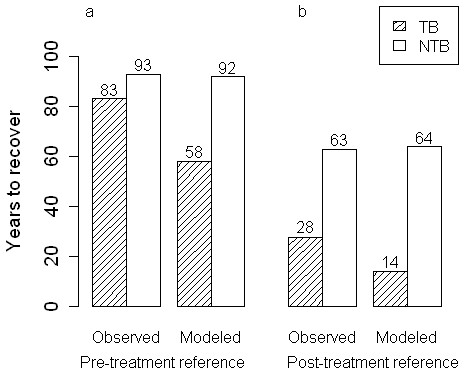
**Carbon recovery timing by treatment, mortality method, and reference baseline.** Time scales of recovery in treated (TB, filled bars) and untreated (NTB, open bars) forest stands, using observed and modeled estimates of mortality rates to set initial conditions, using (**a**) pre-treatment carbon density (175.51 Mg C ha-1) and (**b**) post treatment carbon density (111.85 Mg C ha-1) to define the threshold of recovery. In both observational and simulation based estimates, fuel treated stands in the Angora fire are estimated to recover pre-treatment and post-treatment C stocks more quickly than stands which were not treated for fuels. Because of differences between observed and simulated mortality rates, models parameterized with simulated mortality rates suggest a greater benefit of fuel treatment than using an observationally parameterized model. The choice of a reference point also strongly affects the perceived benefit of fuel treatments on carbon recovery. If post-treatment carbon density is used as a reference point, fuel treated stands are estimated to recover carbon 35 years faster than untreated stands, versus 10 years faster when using pre-treatment C density to define recovery.

Many studies rely on simulated estimates of fire severity when assessing the impact of fuel treatments on long-term carbon stocks. We compared how using observed and modeled estimates of tree mortality during wildfire (Figure 1) might influence the timing of carbon recovery in treated and untreated stands in our study area. Using pre-treatment carbon (175.51 Mg C ha-1) as a baseline, treated stands recover baseline C stocks 10 years more quickly on average than untreated stands (83 vs. 93 years, respectively, Figure 2a), when simulations were parameterized by observed mortality rates. Simulations parameterized by FVS-FFE estimated mortality show treated stands will recover C stocks 34 years more quickly than untreated stands (in 58 vs. 92 years, respectively).

Using post-treatment carbon storage (111.85 Mg C ha-1) as a baseline, TB stands recover baseline C stocks 35 years more quickly than NTB stands (28 vs. 63 years, respectively, Figure 2b) when simulations were parameterized by observed mortality rates. Simulations parameterized by FVS-FFE estimated mortality show TB stands will recover C stocks 50 years more quickly than NTB stands (in 14 vs. 64 years, respectively) using a post-treatment baseline.

### Sensitivity analysis

Simulations of carbon recovery timing in our study area described above suggest that treated stands in the Angora fire may recover pre-disturbance carbon more quickly than untreated stands, at least partially in response to differential rates of tree mortality during wildfire. However, we recognize that these findings are limited to our particular study site. As such, we employed sensitivity analysis techniques to more generally investigate how variation in tree mortality and tree regeneration rates influence expectations of long-term carbon recovery. We used reconstructions of pre-fire forest structure in TB and NTB stands in our study area to represent hypothetical treated and untreated forest stands, and then applied five levels of tree mortality (30% to 100%) and tree regeneration (165 to 1400 seedlings ha^-1^) rates using FVS-FFE, then assessed the impact of this variation on carbon recovery timing.

**Figure 3:**
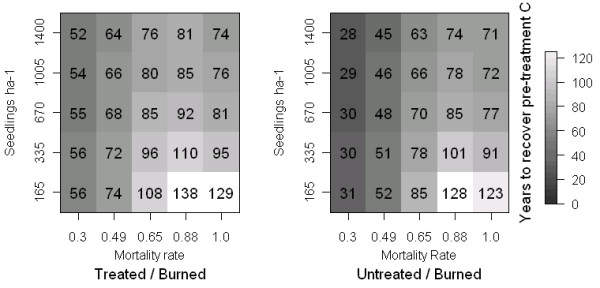
**Carbon recovery timing after wildfire at five levels of mortality and regeneration, untreated reference point.** Years to recover baseline carbon in treated and untreated stands at each combination of five levels of mortality rates and regeneration rates, using pre-treatment carbon storage (175.51 Mg C ha-1) to define the threshold of recovery.

At the start of sensitivity analysis simulations, treated stands contained 36% less C than untreated stands, as estimated at our study site (111.85 vs. 175.51 Mg C ha-1). We estimated that treated stands would recover pre-treatment carbon stocks (175 Mg C ha-1) over a range of 52 to 138 years, while untreated stands required 28 to 128 years to recover (Figure 3). Variation in mortality rates strongly influenced the timing of carbon recovery, regardless of treatment status. Severely burned stands recovered carbon about 20 years more slowly than stands experiencing low mortality rates, when regeneration rates were high. At low rates of regeneration, mortality more strongly influenced the timing of recovery. Stands that were modeled to have low and moderate rates of mortality required 30–60 fewer years to recover C than stands that experiencing mortality rates over 80%.

Regeneration rates were not influential on the timing of recovery at low rates of mortality, but were influential at 65% and higher rates of mortality. Above this level of mortality, stands that had high regeneration rates recovered carbon 30–45 years sooner than sparsely regenerated stands (Figure 3).

**Figure 4:**
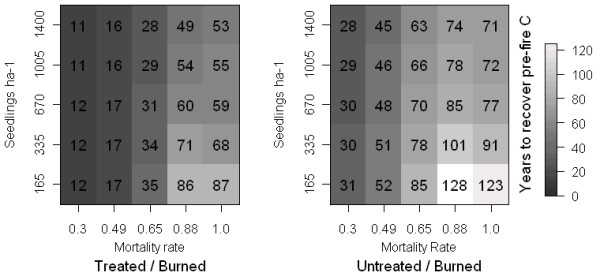
**Carbon recovery timing after wildfire at five levels of mortality and regeneration, treated reference point.** Years to recover baseline carbon in treated and untreated stands at each combination of five levels of mortality rates and regeneration rates, using post-treatment conditions as a reference point (111.81 vs. 175.51 Mg C ha-1 in treated and untreated stands, respectively) to define thresholds of recovery.

We examined how using a different baseline of 112 Mg C ha-1 (post-treatment instead of pre-treatment carbon density) affected our estimates of carbon recovery timing (Figure 4). Treated stands were estimated to recover post-treatment baseline storage in 11 to 87 years after disturbance, depending on the level of regeneration or mortality. This range of recovery times is about 40 years faster compared to using a pre-treatment reference baseline. Regardless of our chosen baseline, recovery times respond strongly to mortality rates, and to regeneration rates when mortality is high.

## Discussion

Our short-term estimates of the direct impacts of fuel treatment and wildfire on aboveground carbon in the Angora burn area show that although these fuel treatments reduced carbon emissions and mortality rates resulting from wildfire, treated stands still stored similar or less overall carbon than untreated stands immediately after fire. This finding corroborates evidence from previous observational [5] and simulation studies [7-10,41], which suggest that C removals during fuel treatment often exceed reductions in pyrogenic C emissions as a result of treatment. Although pyrogenic emissions may be regionally significant sources of atmospheric carbon dioxide [12], fire-related mortality of trees is the single largest carbon transformation that occurs during severe forest fires, carbon which becomes available to future release through decomposition or future fire [12,14,47]. As such, reductions in fire-related tree mortality are thought to be one of the primary mechanisms by which fuel treatments may be able to protect long-term forest carbon stocks, particularly when post-fire regeneration is not sufficiently dense to replace the trees killed during fire [15,26].

In the Angora fire, fuel treatments were effective at reducing multiple measures of fire severity including rates of tree mortality as compared to nearby untreated stands [22]. In part, the effectiveness of the Angora treatments was due to their recent completion, and an extensive treatment prescription that reduced surface, ladder and canopy fuels by removing an estimated 36% of aboveground biomass. Although treated stands stored less carbon than untreated stands before and similar amounts of carbon immediately after wildfire, our long-term simulations suggest that treated stands will recover pre-wildfire carbon stocks 10 to 35 years more quickly than untreated stands, depending on the baseline used. Although this result does not take into account future management or disturbance events, fuel treatments in the Angora fire seem to have been effective at protecting aboveground C stocks over a period of approximately 30–90 years, by proxy of their ability to reduce severe fire effects on vegetation.

Mean FVS-FFE predictions of tree mortality rates at our study site were within +/− 15% of observed rates for both treated and untreated stands. Using modeled mortality rates led to a predicted recovery time in treated stands that was 15 to 25 years longer than when using an observationally parameterized model. This result suggests that assumptions regarding fire severity have a large impact on our assessment of whether fuel treatments could act to protect forest carbon resources over long time scales. Properly implemented fuel treatments are understood to be effective at ameliorating wildfire severity, but observational studies have found a high degree of variation in fuel treatment effectiveness (fire severity) related to variation in treatment prescription and implementation, treatment size, vegetation type, treatment/wildfire timing, and fire weather conditions (see [20]). Previous studies investigating the impact of fuel treatment and wildfire on long-term forest carbon storage account for the influence of a number of these factors, but do not adequately characterize how natural variation in fire severity or post-fire regeneration may influence their findings.

We reviewed five recent studies that used vegetation growth and fire simulation platforms to investigate the long-term impacts of fuel treatment and wildfire upon forest carbon stocks in fire prone forests [7-9,16,48] (Table 4). All five studies reviewed simulated a short-term reduction in stand carbon (range 25% to 43%) due to fuel treatment, in agreement with observational studies of fuel treatments [2,10]. Three of five studies reported that treated stands showed reduced emissions or tree mortality during simulated wildfire events. None of the studies reported that treated stands would store more total C after wildfire, in agreement with observational experiments [5]. Although these studies generally agree about the likely short-term impacts of fuel treatment and wildfire on stand carbon, their long-term predictions are more varied. Over long time scales (100 years), two of the five studies reported that fuel treatments positively influenced carbon storage with benefits limited to forest ecosystems adapted to frequent fire where fire suppression has resulted in uncharacteristically dense forests [7,8]. However, it is difficult to discern whether these simulation studies provide reliable predictions of the long-term effects of disturbance on stand carbon storage, as they do not assess how natural variation regarding fire severity or post-fire vegetation recovery might influence their conclusions regarding long-term C budgets. Specifically, many studies rely on fire simulators to accurately estimate stand-level mortality during wildfire without incorporating variation in fire weather, fuel treatment/wildfire timing or other factors that influence fire severity into their simulation, and do not assess whether modeled rates of mortality are comparable to reference wildfires. To date, most investigators have not reported modeled rates of tree regeneration following wildfire (Table 4) despite evidence that regeneration after wildfire plays an important role in carbon dynamics [14,15,26]. As such, it remains unclear how natural variation in fire-related tree mortality and post-fire vegetation recovery may affect the role of fuel treatments in protecting forest carbon resources.

**Table 4:** Review of studies modeling the impact of fuel treatment and wildfire on long-term forest carbon

**Paper**	**Time scale**	**Growth Model**	**Simulates regeneration?**	**Regeneration rate**	**Simulates mortality in wildfire?**	**Modeled Mortality Rates**
Diggins et al. [38]	100 years	FVS	Yes^1^	N.R.	No^6^	N.A.
Hurteau and North [8]	100 years	FVS	Yes^2^	N.R.	Yes^7^	~ 7-40%^11^
Mitchell et al. [7]	800 or 1600 years	STAND-CARB	N.R.^3^	N.R.	Yes^8^	~10-33%, 45-99%, 60-99%^12^
Reinhardt and Holsinger [9]	100 years	FVS	Yes^4^	N.R.	Yes^9^	~ 14% to 97%
Sorensen et al. [16]	100 years	FVS	Yes^5^	13.934 x e^(−0.022*Basal Area)^	Yes^10^	N.R.

Model parameters and assumptions regarding wildfire-related mortality and post-fire regeneration in five recent studies modeling the impact of fuel treatment and wildfire on long-term forest carbon resources; N.R. = not reported.

^1^ Regen rate from Roccaforte et al. [49] plus 40% (as per Fulé et al. [50]), examines impact of one or two regeneration events in 100 year period.

^2^ Fixed annual rate adapted from Zald et al. [33] (personal communication).

^3^ Did not describe how STANDCARB treats regeneration.

^4^ Uses FVS Regeneration Establishment model defaults for ID/MT (Dixon [33]).

^5^Background rate as function of basal area. Rate from Bailey and Covington [51], 20 year delay after severe fire.

^6^ Simulates prescribed fire with FVS-FFE, not wildfire.

^7^Simulates wildfire with FVS-FFE, extreme fire conditions only.

^8^Simulates wildfire with STANDCARB, using historical fire regimes to set burn frequency and severity.

^9^Simulates wildfire with FVS-FFE, severe fire conditions only.

^10^Simulates wildfire with FVS-FFE, severe fire conditions only.

^11^Percent live tree C killed by wildfire.

^12^Ranges of rates are Expected [Severity] for Coastal range, West Cascades and East Cascades of Oregon, respectively.

### Sensitivity analysis

We found that timescales of carbon recovery after disturbance are highly sensitive to modeled rates of fire-related tree mortality and post-fire regeneration. In our analysis, mortality rates played a particularly strong role in the timing of C recovery. Time until recovery consistently increased with higher mortality rates, regardless of regeneration rate or treatment scenario. Average time scales of carbon recovery increased by 40 years when mortality increased from 30% to 65%, and another 10 years when mortality rates exceeded 90%. This suggests small variations in fire-related mortality rates may have large consequences on the prediction of stand level carbon storage over the next century.

We explicitly applied five levels of fire severity, assuming that stand structure would not affect fire severity in our sensitivity analysis. At equal levels of regeneration and mortality, treated stands always required longer to recover pre-treatment C stocks than untreated stands, as treated stands were assumed to have 36% less biomass at the time of wildfire. However, if fuel treatments are able to reduce rates of wildfire-related mortality, our sensitivity analysis suggests that treated stands could recover baseline C storage more quickly. For example, if treatment led to a reduction in mortality rates from 88% to 49% with a regeneration density of 670 seedlings ha-1, treated stands would recover pre-treatment carbon storage 17 years more quickly than untreated stands (see Figure 3).

After severe fire, our simulations suggest that sparsely regenerated stands may not recover pre-disturbance carbon storage for more than 100 years. After less severe fires, regeneration played a less important role, as surviving trees were responsible for most of the carbon recovery after fire. Although we used a simple model of regeneration, many previous simulation studies have used static or unreported assumptions regarding regeneration. Because our regeneration model only accounted for regeneration in the first year after fire, we also developed two alternate scenarios that allow for regeneration throughout the modeling period beginning 20 years after fire (see Additional file 2). Using alternative regeneration models did not change our general conclusion that regeneration can be an influential parameter on recovery timing, especially when mortality rates are high.

Although wildfires may promote the regeneration of some fire adapted species, severe wildfire may cause temporary or permanent shifts in the structure or composition of vegetation communities [32,35-37]. Factors influencing the potential transition to non-forest vegetation include local extirpation of seed sources [37], post-fire reproductive strategies of local species [35], or competition with rapidly establishing shrubs or grasses [34]. Four years after the Angora fire, there are some indications of a delay or failure of tree regeneration in severely burned stands. In 2011, mean regeneration rates in stands burned by crown fire are only 130 seedlings ha-1, with only 30% of plots containing natural tree regeneration. Severely burned areas also average ~70% surface cover by fire adapted shrubs, and a median distance of over 90 m to the nearest seed source (unpublished data). We acknowledge that these observations may not necessarily signify a transition to a deforested condition, given the short time since disturbance. Nagel and Taylor [36] surveyed six montane chaparral patches created by historical wildfires near our study site. They found that tree establishment in these patches continued for five decades after wildfire, eventually leading to a 62% decrease in the average size of chaparral stands since the advent of fire suppression, due to infill by trees. However, shrubs are vigorous competitors for light and moisture resources [34]. Our sensitivity analysis suggests that a severely burned stand that experiences less dense or slower-growing tree regeneration will require a long period of time to recover carbon, in comparison to a less severely burned site. Although shrubs and non-tree vegetation may be responsible for a large proportion of primary productivity in the first decade after wildfire [11,14], dominance of non-tree life forms combined with the extirpation of nearby seed sources and a changing climate sets up the conditions for a temporary or permanent transition to a non-forest vegetation type [32,37].

### Assumptions and appropriate inference

We made a number of assumptions when modeling mortality and regeneration in our sensitivity analysis. We assumed that mortality rates between 30% and 100% were possible for both treated and untreated stands, and did not model the effect of stand structure or fuel treatment on fire severity in this portion of our analysis. When modeling regeneration we assumed that tree seedling densities would not co-vary with modeled mortality rates and decided not to consider the possibility of deforestation or delayed reforestation. Despite evidence that non-tree vegetation can be responsible for a substantial proportion of carbon uptake in the first decade after fire, particularly in severely burned forests [11,14], we did not consider these dynamics as understory vegetation is not well modeled by FVS. We focused on the role that tree regeneration density plays in carbon budgets and did not assess how variation in other stand characteristics (e.g. species composition or age structure) might affect our conclusions. Although we only examined how variation in one aspect of succession (tree density) is influential on carbon recovery, few other studies have explicitly examined how variation in mortality or post-fire regeneration influence predictions of stand level carbon budgets.

Our analysis evaluated how variation in mortality and regeneration influence carbon recovery over one fire return interval, without considering how future disturbance or management regimes might affect carbon dynamics. Before Euroamerican settlement, forests in our study area supported a high frequency/low severity fire regime with fire return intervals ranging from 5–30 years with a mean return interval of ~11 years [52-54]. Under pre-settlement fire regimes, 4 to 14 fires would have been expected to burn in the study area over the temporal course of our longest recovery scenarios. Although human fire suppression has succeeded in excluding fire from most of the Lake Tahoe Basin for a century, recent fires have been bigger and more difficult to suppress. For example, all fires >200 ha in size that occurred over the last 100 years have occurred in the last decade. Future climate and fire projections under global warming and increasing human population densities suggest that fire risk will rise significantly over the next century (e.g. [55,56]), and indeed current increasing trends in fire activity, area, and severity in the Sierra Nevada suggest that such changes are already underway [57]. A further issue is future fuel treatment plans in the study area, which falls almost entirely within mapped Wildland-Urban Interface (WUI). Treatment effectiveness in Sierra Nevada yellow pine or mixed conifer forests decreases substantially after about 10 years [58]. Forest Service strategies for long-term management often assume treatment re-entries on a rotation of at least 20–30 years. But as with wildfire, our scenarios were not able to account for the potential effects of these recurrent future biomass removals.

### Implications for management

In many Western forests, fire suppression has allowed biomass to accumulate beyond what would be expected under naturally occurring fire regimes [24,59,60]. However, the carbon stored in uncharacteristically dense forests may be at risk if stand replacing wildfire occurs, due to large emissions resulting from fire and the potential for changes in vegetation type [17]. In the debate over whether fuel treatments are an appropriate management strategy to protect forest carbon resources, a number of studies have focused upon the ability of fuel treatments to mitigate increases in atmospheric carbon, by either reducing emissions resulting from wildfire, or by storing more carbon as compared to untreated stands [4,40,61]. However, given the unpredictable nature of wildfire and the recurrent biomass removals required to effectively reduce wildfire risk, a number of studies agree that fuel treatments may be an ineffective climate mitigation strategy unless treated biomass is used in other carbon positive activities (i.e. wood products or energy generation, [9,10,23,24])

Hurteau and Brooks [3] proposed that fuel reduction treatments may be better characterized as adaptive management tools that aid in stabilizing existing forest carbon stocks under a natural disturbance regime (carbon carrying capacity, [62]). If we decide that maintaining a fire-resistant forest structure through fuel reduction is an appropriate strategy to promote stable (but not maximal) forest carbon storage, identifying a carbon carrying capacity appropriate for the forest in question will be an important task [24]. We found that using a post-treatment carbon baseline (Figures 2 and 4) to define carbon recovery increased the perceived benefit of fuel treatments. When we used post-treatment C stocks (112 and 175 C ha-1 in treated and untreated stands, respectively) to define baseline conditions, we found that treated stands recovered pre-fire C more quickly even if treatments did not reduce mortality rates during wildfire. If treatments do reduce wildfire related mortality from 88% to 49%, our simulations suggest that treated stands recover baseline C five times faster than untreated stands (17 vs. 85 years). Although the treated stands may not necessarily store more carbon than untreated stands at any given point when we assume two different baselines, treated stands may tolerate a number of intermediate disturbances in the same time period that it takes an untreated stand to recover from a single severe disturbance. This finding coincides with the theoretical framework provided by Hurteau and Brooks [3], who illustrate potential tradeoffs between managing for stable and maximal carbon stocks in fire prone forests.

Forest management activities such as thinning, prescribed burning, logging, or replanting after fire are resource intensive. Our analysis demonstrates that reducing mortality during future wildfire events should be a key goal of fuel treatments, if carbon storage is a long-term management goal. If severe fire does occur, regeneration monitoring and tree planting will be important to ensure prompt recovery of carbon stocks. Fuel treatments may result in increased growth or increased reproductive output among the remaining trees, and may enable treated forests to avoid future drought- or insect-related mortality by reducing stress on trees due to competition for water or light [63,64]. Of course, long-term carbon storage is not the only critical consideration for resource managers. There is likely a tradeoff between regeneration or reforestation resulting in densely stocked forests and future fire risk, that must be navigated with future multiple resource objectives in mind [65]. The species composition and age structure of post-fire regeneration may also be highly important to managers seeking to maintain fire-resistant forest communities dominated by pines. Similarly, a focus on minimizing mortality may come at the cost of fire-obligate species [66].

## Conclusions

Our ability to understand how anthropogenic and natural disturbances affect forest carbon resources hinges on our ability to adequately represent processes known to be important to long-term forest carbon dynamics. At our study site, treatments removed more biomass than was saved through reductions in pyrogenic emissions due to treatment. However, differences in tree mortality rates and regeneration rates between treated and untreated stands were such that treated stands are projected to recover pre-disturbance carbon storage more quickly than untreated stands. More generally, we showed that assumptions regarding rates of fire-related mortality strongly influence our understanding of long-term forest carbon dynamics at all levels of fire severity. Assumptions regarding post-fire tree regeneration were also found to be influential, but much more so after severe wildfire. We recommend that uncertainty in disturbance severity, disturbance recovery, or other influential parameters be more carefully considered in future efforts to model carbon dynamics in forests affected by wildfire. To the extent that fuel treatments are able to reduce tree mortality rates during fire, or encourage post-fire tree regeneration, our analysis shows that fuel treatments could be a viable strategy to promote more rapid recovery of pre-existing forest carbon stocks in the forest type we studied.

## Methods

### Study site description

**Figure 5:**
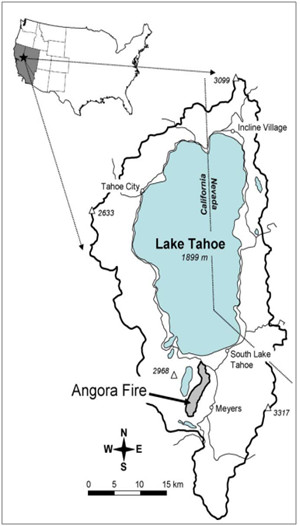
Location of Lake Tahoe Basin and the Angora Fire.

The Angora fire is located within the Lake Tahoe Basin (LTB), in the Sierra Nevada of California and Nevada (Figure 5). Elevations in the basin range from 1800 m to 3315 m at Freel Peak. The climate is Mediterranean, with warm dry summers and cold wet winters. At the South Lake Tahoe, CA airport (1900 m elevation, 3 km E of the Angora Fire), the January mean minimum temperature is −10.4°C, July mean maximum is 23.5°C. Precipitation averages 784 mm per year, with 86% of precipitation falling as snow between November and April [67].

#### Forest fires in the Lake Tahoe Basin

Pre-settlement fire return intervals in the Tahoe Basin were 5–30 years in *Pinus jeffreyi* dominated forests and 20–45 years in upper montane forests dominated by *Abies magnifica*[36,53,54,68]. Between 1873 and 1900, most LTB forests (including our study site) were heavily logged or clearcut and extensively grazed until the 1930s [68,69]. Over the last century, active fire exclusion in the LTB has nearly eliminated fire as a natural process. The history of logging and fire exclusion has resulted in increases in tree density, canopy cover, and surface fuels in many areas [68,70]. Before the Angora fire, only three sizable natural wildfires have occurred in the LTB in the last 100 years, largely due to effective fire suppression [22].

#### The Angora fire

On June 24, 2007 the Angora wildfire was ignited from an illegal campfire and burned 1106 forested hectares (1243 total ha) over eight days. The Angora fire burned early in the fire season, under record dry conditions for that date [71]. More than half of the burn area experienced >75% tree mortality according to remotely sensed estimates of burn severity. About two-thirds of the fire burned in the first day, after which winds moderated and shifted to the north.

Elevations in the Angora fire range from 1900 m on the northern boundary to 2310 m on the SW boundary. Soils are generally coarse textured and well drained. Geologic substrates are primarily granitic, with some metamorphic formations. Slopes range from 0–5% along the Angora creek drainage to >40% along the western and southwestern borders of the fire.

Vegetation is primarily conifer forest with Jeffrey pine (*Pinus jeffreyi*) and white fir (*Abies concolor*) dominating lower slopes, and red fir (*Abies magnifica*) primarily occurring on slopes above 2100 m. Incense cedar (*Calocedrus decurrens*), sugar pine (*P. lambertiana*), lodgepole pine (*P. contorta var. murrayana*) and Quaking aspen (*Populus tremuloides*) are also present in minor amounts, with the latter two species concentrated along drainages. Montane chaparral is found on east-facing slopes along the south and western boundaries of the fire and in scattered patches elsewhere, dominated by *Arctostaphylos patula, Quercus vaccinifolia, Chrysolepissempervirens* and species of *Ceanothus*. The last recorded fire in the Angora area was a wildfire in 1882 [36], which overlapped with areas of montane chaparral and white fir forest burned by the Angora fire.

#### Fuel treatments in the Angora fire area

**Figure 6:**
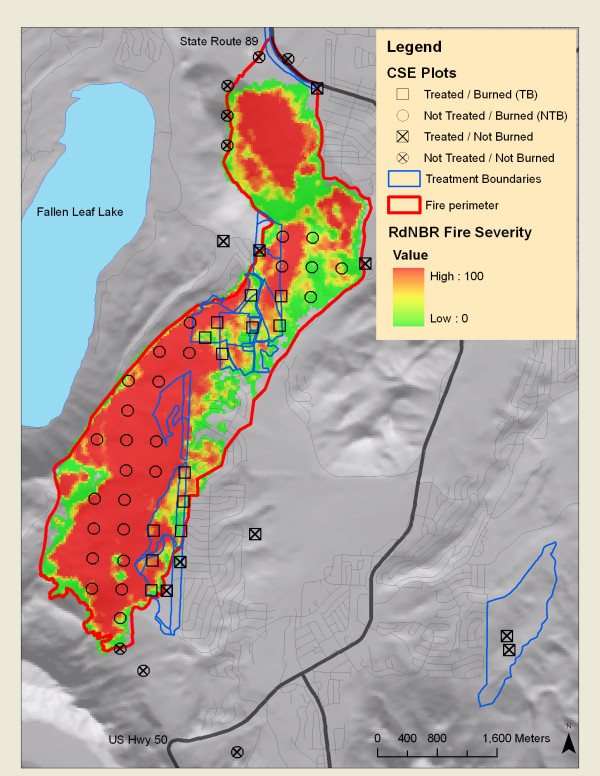
**Map of the Angora fire.** Remotely sensed map of fire severity (RdNBR), overlaid with positions of Common Stand Exam (CSE) plots used in analysis. Plots located in Treated and Burned stands (TB, blue outlines) are marked with an open square (n = 13), plots located in stands which were Not Treated and Burned (NTB) are marked with an open circle (n = 26). Treated and untreated plots sampled outside the fire are marked with filled circles and squares, respectively (n = 9 and n = 9, respectively).

Approximately 182 ha (16%) of the burn area had been treated for fuels between 1996–2006 (Figure 6). Treatments generally consisted of a pre-commercial hand thin, a commercial thin and ‘salvage’ of standing dead material, followed by hand piling and burning. Mechanical thinning prescriptions called for a residual basal area of 36.7 m^2^ ha^-1^ for trees >25.4 cm DBH in mechanically thinned stands, and snags less than 76.2 cm diameter were cut. Hand thinning left all trees greater than 35.6 cm DBH, and removed smaller trees to achieve an average bole spacing of 6.1 m. Crews were instructed to hand pile all thinning residues, as well as undecayed coarse woody debris (for a complete description of treatment prescriptions see [22,71]). Pre-fire fuel loadings in the Angora fire were estimated at 11 tons biomass ha^-1^ in treated stands and 57.9 tons ha^-1^ in neighboring untreated forest [22].

### Sampling and measurements

#### Plot selection procedures

For three subsequent summers after the Angora fire, we established and surveyed eighty-six permanent vegetation plots in and around the wildfire on a 400-m grid using USFS Region 5 Common Stand Examination (CSE) protocols detailed below [72]. We use a subset of these plots in our analysis, selecting only plots that burned within the first twenty-four hours of fire ignition (Figure 6). Sixteen plots were located in stands that had been treated before wildfire (TB stand). We excluded three of sixteen TB plots from analysis because they were located in a treatment unit where piles had not been burned before the fire and where logging occurred after the fire, for a total of 13 TB plots. Twenty-nine plots were located in stands that were not treated before burning in the wildfire (NTB stand) and were within 800 m of treated stands. Three NTB plots located in densely stocked riparian areas dominated by *P. contorta* and *P. tremuloides* were excluded from analysis for a total of 26 NTB plots. Treated and untreated stands were identified using a GIS layer of treatment history obtained from Lake Tahoe Basin Management Unit staff and field verifying the maps with observations of recently cut stumps (as per [22,71])

We also surveyed nine treated and nine untreated plots just outside the wildfire using CSE protocols. We used fuels information from plots outside the fire to estimate fuel loading in treated and untreated stands before wildfire. Most unburned plots were adjacent to the fire, but we were forced to sample five plots (3 TB, 2 NTB plots) outside of the immediate vicinity of the fire because of the lack of comparable forest area. We used previously established USFS plots when possible, and identified treatment history using USFS treatment records and field verification. Unburned plots were all located within a few kilometers of the fire, and selected based on their age, density, and species composition. Given the clearcut logging that occurred throughout the LTB in the 1890’s, forest stands in this area have a similar age, species composition and forest structure, so estimates of fuel loading from outside the fire should be representative of pre-fire fuel conditions in treated and untreated stands.

Two hundred “regeneration” plots were also established on a 200-m grid across the fire. Each CSE plot had a co-located regeneration plot at its center. Regeneration plots that were logged after fire were removed from the analysis, leaving 37 and 71 plots located in treated and untreated stands, respectively.

#### Field protocol

##### Common Stand Exam plots

CSE plots were circular, with an area of 809.37 m^2^ (16.06 m radius, equal to 1/5 acre). In 2008 (one year after fire) we tagged live trees above a breakpoint of 12.7 cm DBH, and snags above 25.4 cm DBH on each CSE plot. For each above-breakpoint tree and snag in 2008, we recorded the species, diameter, pre- and post-fire mortality status and post-fire live crown ratio. A subset of tree heights (first five mature trees on each plot) was recorded. Above-breakpoint trees and snags in the burn area were revisited in 2009 and 2010, when we recorded further mortality, insect/disease damage, stem breakage, or tree cutting. Trees below the breakpoint were counted and tallied by species, mortality status, and diameter (2.54 to 12.7 cm and 12.7 to 25.4 cm DBH). Because we were unable to determine whether dead small trees had been alive or dead before the fire, we assumed they were alive. Although this could upwardly bias our estimates of pre-fire live tree carbon, it will also downwardly bias estimates of pre-fire snag carbon. We tallied stumps in 12.7 cm size classes on each plot to assess thinning impacts on tree carbon.

Surface woody fuels were surveyed on CSE plots using standard planar intercept protocol [42,43]. On each plot visit, we surveyed four 15.24 m fuels transects radiating from plot center in four cardinal directions. On all four transects, we counted fuels <0.64 cm and 0.64–2.54 cm diameter along a total of 12.19 m and fuels 2.54–7.62 cm diameter along 30.48 m, beginning at the distal end of the transect. We recorded the diameter and decay class of logs >7.62 cm diameter for any piece >1 m in length that intersected any transect. We recorded additional log measurements in 2010, including small and large end log diameters and log length [42]. We also took two litter and duff depth measurements on each fuels transect, for a total of eight depth measurements per plot.

##### Regeneration plots

We surveyed regeneration plots in the summer of 2008, and re-visited these plots in 2009 and 2010. At each 60 m^2^ circular plot we tallied tree seedlings by species and age, separately counting planted, natural, and pre-fire regeneration. Seedlings were identified to species using an identification guide [73].

At each regeneration and CSE plot, we assigned a plot-wide categorical severity class (1–5) based on guidelines-related to fire effects on trees and vegetation. A severity rating of 5 denotes sustained crown fire across the plot, a rating of 4 indicates high mortality but no sustained crown fire, a rating of 1 indicates a ground fire that incompletely consumed surface vegetation and killed few trees, while a rating of 2 or 3 represents intermediate levels of fire severity and mortality (adapted from [74]).

### Short-term impact of treatment and wildfire on C pools

We estimated the carbon density (Mg C ha-1) of five aboveground biomass pools (live trees, snags, coarse woody debris, fine woody debris, litter/duff) at five time steps (pre-treatment, pre-wildfire, 2008, 2009, 2010) in treated and untreated stands in our study area, using published allometric equations implemented in the Forest Vegetation Simulator Western Sierra variant (Table 1). To estimate pre-thinning and pre-fire C storage, we used indirect methods because we did not survey plots before wildfire. Specifically, we used fuels data from unburned plots to estimate pre-disturbance C stocks, and stump surveys and observations of tree mortality during fire to reconstruct pre-thinning and pre-wildfire tree lists. We directly estimated the density of C pools after fire, using observations from CSE plots. We used a two-sided Wilcoxon rank sum test to test for differences in total aboveground C and component C between TB and NTB stands at each time step, using the statistical analysis software R [75]. See Additional file 2 for further details on biomass estimates.

### Long-term impact of treatment and wildfire on C pools

A secondary objective of this study was to assess how fuel treatments and wildfire impacted long-term carbon resources in treated and untreated stands at our study site. We used observations of tree mortality, tree regeneration, and forest structure made in TB and NTB stands in 2010 (three years after wildfire) to initialize FVS, grow the stands forward, and calculate the years elapsed before stands recovered baseline carbon stocks. We then assessed how using a fire simulator to predict tree mortality might influence our findings. To do so, we repeated the same steps as above, but used a model (FVS-FFE) instead of observational data to predict tree mortality rates during wildfire. We parameterized the fire model using reconstructions of pre-fire stand conditions and fuel moisture and fire weather conditions recorded during the day of the fire (Additional file 2, [71]).

The same regeneration rates were applied to each model run (observed and modeled mortality), using observations made at regeneration plots co-located with the 13 treated and 26 untreated plots used in analysis. Naturally occurring regeneration rates in the 13 treated and 26 untreated CSE plots used in these simulations averaged 479.2 and 148.8 seedlings ha-1, respectively. If no regeneration was present in 2010, we added 165 white fir seedlings ha-1 twenty years into the simulation to avoid simulating deforestation.

### Sensitivity analysis

We assessed how variation in two key ecosystem processes (tree mortality and regeneration) influenced years until carbon recovery after wildfire in treated and untreated stands. We used pre-treatment carbon density in untreated stands as our primary reference point for estimating years until recovery, as it represents baseline forest conditions before carbon losses due to fuel treatment or wildfire. However, we recognize that post-treatment conditions may be a more appropriate target for management (sensu Hurteau and Brooks [3]), and explored how the use of an alternate reference point (post-treatment carbon density) affects our results. To accomplish our sensitivity analysis, we initialized FVS with reconstructions of pre-fire (post-treatment) stand conditions in TB and NTB stands. We then simulated five levels of fire-related mortality and five levels of post-fire regeneration, using information collected in our study area to bound these variables as described below. We used FVS to simulate mortality, regeneration, decay, and growth over a 150 year time period, and calculated the time required to recover pre-treatment (and post-treatment) carbon stocks at each level of mortality and regeneration, for treated and untreated stands. FVS reports stand level metrics on 10 year time steps, so we used linear interpolation to estimate a specific year of recovery.

#### Mortality

**Figure 7:**
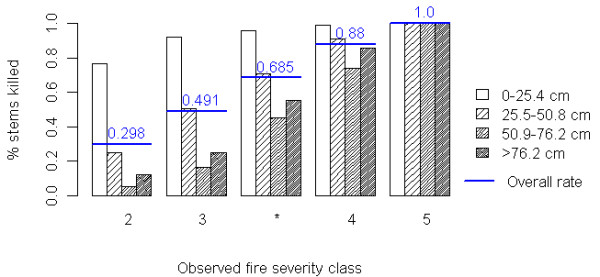
**Mortality rates used in sensitivity analysis.** Mortality rates by diameter class used to define each of five mortality scenarios used in sensitivity analysis. Mortality rates in scenarios 1,2,4 and 5 were directly estimated from CSE plots in field assigned fire severity classes 2,3,4 and 5. We decided to create a central fifth mortality class (with an overall mortality rate of 68.5%) by averaging observed mortality rates in severity classes 3 and 4, to avoid a large discontinuity in our sensitivity analysis.

We used observations of mortality rates from the 39 CSE plots in our study area to define five mortality scenarios for sensitivity analysis. We pooled estimates of tree mortality rates by diameter class for all the CSE plots in each of four categorical fire severity classes to create four mortality scenarios (Figure 7). We created a central fifth mortality scenario by averaging mortality rates from severity categories 2 and 3, in order to model a full range of mortality rates (Figure 7). Each of the five mortality scenarios defines a different mortality rate for four tree diameter classes (0–25.4 cm, 25.5 cm to 50.8 cm, 50.9 to 76.2 and 76.3 + cm), based on data from our study site. Overall mortality rates range from 30% in the least severe scenario to 100% mortality in the most severe. We recognize that more intense fires are associated not only with higher tree mortality, but also produce more emissions from surface carbon pools [12,14]. Each of five mortality scenarios had an associated set of combustion factors that were applied to pre-fire surface carbon pools (fine woody debris, coarse woody debris, litter and duff) to differentiate between the impact of less and more severe wildfire on stand carbon storage (see Additional file 1 for rates). We used previous research as the basis for setting our combustion co-efficients [12].

#### Regeneration

**Figure 8:**
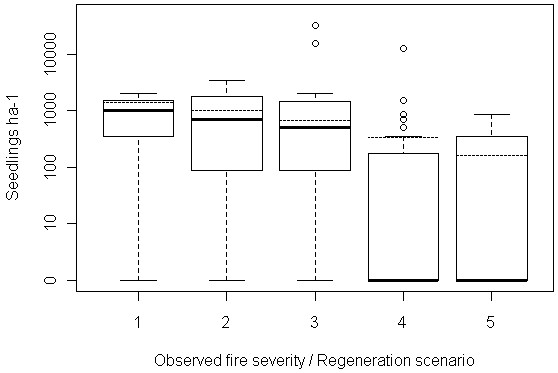
**Regeneration rates used in sensitivity analysis, with observed rates.** Density of natural conifer regeneration (log scale) observed three years after the Angora fire in five categorical fire severity classes (boxplots), overlaid with regeneration rates used in sensitivity analysis models (dashed lines). We varied regeneration rates at one of five densities (1400, 1005, 670, 335, and 165 seedlings ha-1) in our sensitivity analysis. These rates were chosen to represent a realistic range of post-wildfire forest densities, consistent with re-constructions of pre-wildfire live tree densities at our study site (197 to 1754 trees ha^-1^).

We chose to represent variation in regeneration using a simple model, where we varied post-fire regeneration at one of five densities between 165 to 1400 seedlings ha^-1^, split evenly between Jeffrey pine and white fire, and did not add additional regeneration during the simulation period. These regeneration rates were selected by varying the median seedling density of all plots containing regeneration (670 seedlings ha-1) by plus or minus 50% and 75% (Figure 8). The five regeneration rates chosen fell within the range of pre-disturbance forest densities reconstructed at our study site (197 to 1754 trees ha^-1^, median ~800 trees ha^-1^). We explored how allowing for additional regeneration throughout the modeling period influenced our results (Additional file 2), and found that the use of more sophisticated regeneration models did not change our general conclusions.

## Abbreviations

TB, Treated and burned; NTB, Not treated and burned; CSE, Common Stand Exam; DBH, Diameter at Breast Height; FVS, Forest Vegetation Simulator; FVS-WS, Forest Vegetation Simulator Western Sierra Variant; FVS-FFE, Forest Vegetation Simulator Fire and Fuels Extension; C, Carbon; FWD, Fine Woody Debris (surface fuels < 7.62 cm in diameter); CWD, Coarse Woody Debris (surface fuels > = 7.62 cm in diameter); NEP, Net Ecosystem Production.

## Competing interests

The authors do not have any competing interests to declare, other than those stated in our institutional affiliations. This article has not been previously been published in or submitted to a peer reviewed journal, although portions were used to satisfy the thesis requirement for an M.S. in Forestry at the University of Montana College of Forestry and Conservation. The thesis will be available on the web in February 2012 via the Mansfield Library website. Some of the data used in this article were used in a 2009 Forest Ecology and Management article investigating the impact of fuel treatments on wildfire behavior [22], but the scope of the 2009 paper does not include forest carbon, and we avoid repeating any statistical tests for differences in pre- or post-fire forest structure, or measures of fire severity.

## Authors’ contributions

CHC led field sampling, developed the methodology, conducted simulation modeling, performed statistical analyses, and did most of the writing. SZD conceived the study, assisted with designing methods and interpreting results, and edited the manuscript. HDS helped design and implement field sampling, assisted with interpretation and drafting of the manuscript. All authors read and approved the final manuscript.

## Supplementary Material

Additional file 1 ** Pre- and post-fire carbon estimates.** Data table containing: estimates of carbon pool sizes, regeneration rates, and predicted and observed mortality rates, for 13 treated and 26 untreated Common Stand Exam plots located in the Angora fire. Click here for file

Additional file 2 ** Appendix A.** Additional information regarding biomass estimates, wildfire simulation settings, and regeneration scenarios. Click here for file
