# Supplementary material for: Variation in tree mortality and regeneration affect forest carbon recovery following fuel treatments and wildfire in the Lake Tahoe Basin, California, USA
Source: Carbon Balance Manag. 2012 Jun 28;7:7. doi: 10.1186/1750-0680-7-7 (PMC3430563; doi:10.1186/1750-0680-7-7)
Supplement: Additional file 2 — Appendix A. Additional information regarding biomass estimates, wildfire simulation settings, and regeneration scenarios. [file 1750-0680-7-7-S2.doc]

**Additional File 1**

**Appendix A**

**1.0 Carbon pool estimation**

We estimated the C density (measured in Mg C ha^-1^) of the following five pools at five time steps for each of 39 plots in our study area: live trees, dead trees, fine woody debris (FWD, < 7.62 cm diameter), coarse woody debris (CWD, ≥7.62 cm diameter), and litter and duff. For the purpose of this study, the sum of these five pools is equal to aboveground C. Although understory vegetation, soil carbon and root carbon may constitute a substantial proportion of forest carbon stocks, we did not include them in our analysis for a variety of reasons. These pools are not required by many forest carbon protocols, are not well modeled in the Forest and Vegetation Simulator (FVS [1]), and are minimally affected by wildfire over the time period of interest.

We estimated the carbon contained in live and dead trees with the Western Sierra variant of FVS. FVS uses species specific volume equations and wood density values to estimate live and dead tree biomass, and a factor of 0.5 to convert wood biomass into carbon. We used biomass equations for *Abies concolor* to estimate the carbon contained in trees which were removed during thinning, as we did not separate our stump surveys by species, and *Abies concolor* was the most commonly removed tree species.

We estimated the carbon content of surface fuels (FWD, CWD, litter/duff) by first estimating fuel biomass using conventional methods and then converting biomass into carbon using a factor of 0.5 for woody fuels, and 0.37 for litter and duff [1]. Biomass of FWD was estimated using techniques set forth in Brown [2], using published estimates of average piece diameter and secant, averaged for Jeffrey pine and white fir fuelbeds [3]. Biomass of CWD was estimated as per Waddell [4]. Litter and duff biomass was estimated using published allometries relating litter duff biomass as a simple function of depth. We averaged the co-efficients for *P. jeffreyi* and *A. concolor* fuelbeds, coming up with the equation: where BM_LD_ is biomass of litter/duff in kg m^-2^ and depth is measured in cm [5].

**2.0 Sensitivity analysis**

**2.1 Additional description of mortality scenarios:**

We developed five different mortality scenarios using field based estimates of tree mortality rates by diameter class to define each scenario. We recognize that fires which kill more trees will also likely consume a greater proportion of existing biomass. We attempted to account for this by using a simple set of combustion co-efficients to model increasing consumption of surface carbon pools by more intense fire. We used estimates of combustion rates by carbon pool from Campbell et al. [6] as the basis for our simple model (Appendix A Table 1).

**2.2 Additional description of regeneration scenarios**

Although we only present results from our most simple regeneration model, we also examined how using a more sophisticated regeneration model might influence our results.

In the model we presented (regeneration model A), we simulated five different densities of regeneration (1400, 1005, 670, 335, and 165 seedlings ha-1) immediately after fire, splitting seedlings between Jeffrey pine and white fir, and did not add additional regeneration throughout the modeling period. Although this model results in a range of forest densities, it does not account for the impact of continued regeneration upon time scales of recovery.

We developed two additional regeneration models (models B and C) that use the same 5 rates of post-fire regeneration used in model *A* (165 to 1400 seedlings ha-1) in the first 20 years after fire, but also add additional regeneration throughout the modeling period. In models B and C, initial regeneration is prolonged over 20 years, with 50% of regeneration applied in year 1 after fire, 25% in year 5, 15% in year 10, and 10% in year 20.

In regeneration model B, continuing regeneration is based on the basal area of Jeffrey pine and white fir in the simulated stand. Every decade starting 20 years after fire, we added 300 white fir seedlings ha-1 per m2 ha-1 basal area of mature white fir in the stand, and 100 Jeffrey pine seedlings ha-1 per unit basal area mature of Jeffrey pine, values based on mean seedling establishment rates per pre-fire basal area of these two species in our study area (data not shown). Survival rates were set as 5% and 10% for *A. concolor* and *P. jeffreyi*, similar to rates reported in the literature [7]. In this model, stands which experienced a lower fire-related mortality received more continuing regeneration, due to the higher basal area of mature trees (i.e. ‘rich get richer’ regeneration model).

In regeneration model C, we simulate regeneration as a fixed decadal amount, regardless of overstory basal area, adding 300 white fir and 100 Jeffrey pine seedlings every 10 years starting 20 years after fire, with a 100% survival rate.

**2.3 Impact of using different regeneration models on sensitivity results:**

Using regeneration model B in our sensitivity analysis, we predicted similar time scales of recovery for most combinations of regeneration and mortality. In simulations where where mortality was high, and initial regeneration low, the use of Regen model B bresulted in carbon recovery about 10 years faster than in our simplest model. (Appendix A Figure 1).

Regeneration model C added significantly more regeneration throughout the modeling period than either model A or B (161.9 trees ha-1 decade-1 after 20 years). Using this model did not significantly change our results. In simulations where where mortality was high, and initial regeneration low, the use of Regen model C resulted in carbon recovery about 25 years faster than in our simplest model. However, initially low regeneration still clearly influences time scales of recovery, and mortality becomes an even more dominant of a driver of time scales of recovery (Appendix A Figure 1).

**3.0 Forest Vegetation Simulator Settings**

**3.1 Wildfire modeling**

We used the Fire and Fuels extension to FVS (FVS-FFE, [8]) as a tool to compare how estimates of carbon recovery after disturbance might differ when using modeled versus observed tree mortality at our study site. We simulated wildfire in treated and untreated stand, using reconstructions of pre-fire conditions, and fire weather conditions observed during the Angora wildfire. Murphy et al. [9] report that on the day that the Angora fire ignited, large dead fuel moisture was 9%, life woody fuel moisture was 73%, minimum relative humidity was 8%, and wind gusts ranged from 5 to 22 miles per hour, with firefighters reporting stronger gusts. FVS-FFE requires more inputs regarding fuel moisture and fire weather than were reported by Murphy et al. [9]. We used the following parameter settings in our wildfire simulation, using defaults for “severe” wildfire where published estimates were not available (Appendix A Table 2).

**3.2 Forest growth modeling**

We used appropriate default FVS settings for our study site. We used a site index of 50, an elevation of 6500 ft, and selected Jeffrey pine as a site species. We used annual decay rates of 0.25 for litter, 0.002 for duff, 0.025 for fuels 0-4.62 cm diameter, and 0.013 for fuels >4.62 cm diameter (FVS-WS defaults). When we added post-fire regeneration using the PLANT keyword, we used a 100% establishment rate (any mortality was simulated by the density dependant model).

**References**

1. Dixon GE, comp.: **Essential FVS: A user’s guide to the Forest Vegetation Simulator**. Internal Report, USDA Forest Service, Forest Management Service Center; 2002. [<http://www.fs.fed.us/fmsc/ftp/fvs/docs/gtr/EssentialFVS.pdf>].

2. Brown JK: **Handbook for inventorying downed woody material**. *GTR-INT-016.* USDA Forest Service, Intermountain Forest and Range Experiment Station; 1974.

3. van Wagtendonk JW, Benedict JM, Sydoriak WM: **Fuel Bed Characteristics of Sierra Nevada Conifers**. *WJAF* 1998, **13**:73-84.

4. Waddell KL: **Sampling coarse woody debris for multiple attributes in extensive resource inventories**. *Ecological Indicators* 2002, **1**:139-153.

5. van Wagtendonk JW, Benedict JM, Sydoriak WM: **Physical Properties of Woody Fuel Particles of Sierra Nevada Conifers**. *Int J Wildland Fire* 1996, **6**:117-123.

6. Campbell J, Donato D, Azuma D, Law B: **Pyrogenic carbon emission from a large wildfire in Oregon, United States**. *J Geophys Res* 2007, **112**:1-11.

7. Zald HSJ, Gray AN, North M, Kern RA: **Initial tree regeneration responses to fire and thinning treatments in a Sierra Nevada mixed-conifer forest, USA**. *For Ecol Manage* 2008, **256**:168-179.

8. Rebain, SA, comp.: **The Fire and Fuels Extension to the Forest Vegetation Simulator: Updated Model Documentation.** Internal Report, USDA Forest Service, Forest Management Service Center; 2010. [<http://www.fs.fed.us/fmsc/ftp/fvs/docs/gtr/FFEguide.pdf>].

9. Murphy K, Rich T, Sexton T: **An Assessment of Fuel Treatment Effects on Fire Behavior, Suppression Effectiveness, and Structure Ignition on the Angora Fire**. *R5-TP-025.* USDA Forest Service, Lake Tahoe Basin Management Unit; 2007.

**Figures**

**Appendix A Figure 1a-1c.** Sensitivity results using regeneration model A, B, and C.


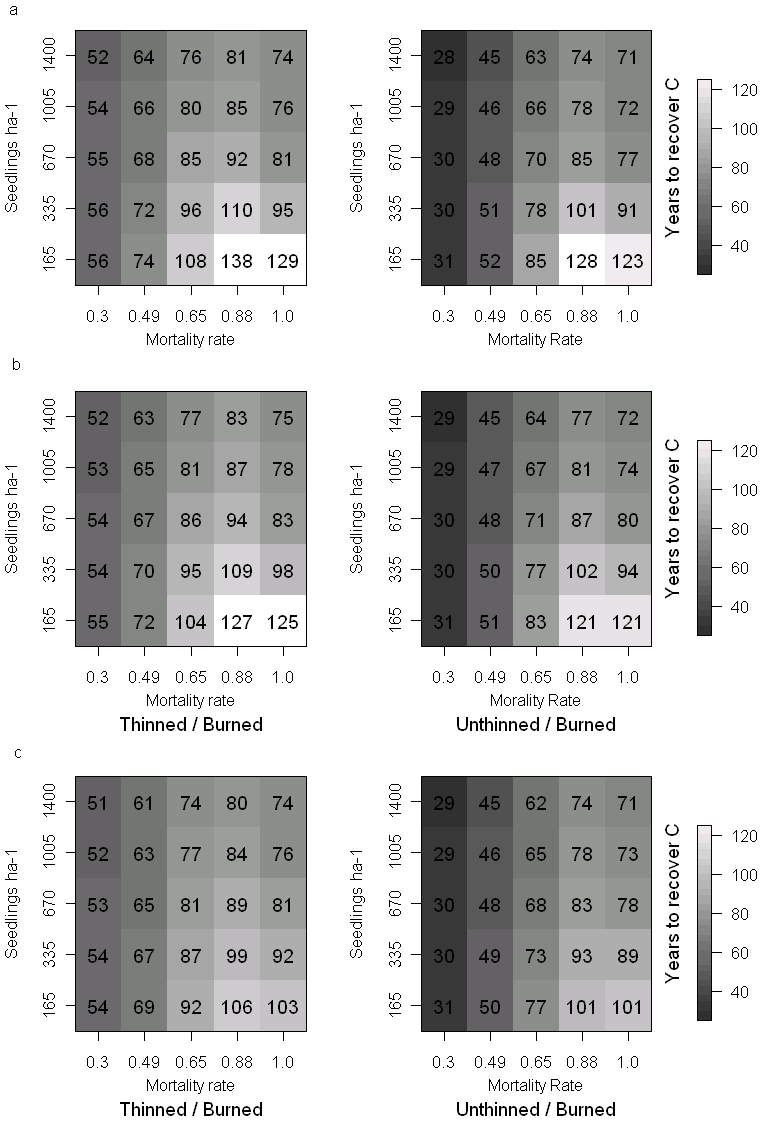


**Tables**

| **Mortality Scenario** | **% LD consumed** | **% FWD consumed** | **% CWD consumed** |
| --- | --- | --- | --- |
| 1 | 0.6 | 0.6 | 0.4 |
| 2 | 0.7 | 0.7 | 0.5 |
| 3 | 0.8 | 0.8 | 0.6 |
| 4 | 0.9 | 0.9 | 0.7 |
| 5 | 1.0 | 1.0 | 0.8 |

**Appendix A Table 1.** Combustion rates as a percentage of pre-fire mass for litter and duff (LD), fine woody debris <7.62 cm diameter (FWD) and coarse woody debris > 7.62 cm diameter (CWD), for mortality scenarios 1-5. Values based on Campbell et al. [6].

| Parameter | Value |
| --- | --- |
| Windspeed | 20 mph* |
| 1 hr (% moisture) | 10 |
| 10 hr (% moisture) | 10 |
| 100 hr (% moisture) | 10 |
| 3”+ (% moisture) | 9* |
| Duff (% moisture) | 152 |
| Live woody (% moisture) | 73* |
| Live herb (% moisture) | 150 |

**Appendix A Table 2.** Settings used during simulation of wildfire mortality, using the Forest Vegetation Simulator Fire and Fire Effects (FVS-FFE) extension. Values marked with a * indicate values taken from observations made at the time of the wildfire [9], with all other values representing default FVS-FFE “severe” fire weather conditions.
